# Prefrontal event-related potential markers in association with mild cognitive impairment

**DOI:** 10.3389/fnagi.2023.1273008

**Published:** 2023-10-19

**Authors:** Joel Eyamu, Wuon-Shik Kim, Kahye Kim, Kun Ho Lee, Jaeuk U. Kim

**Affiliations:** ^1^Digital Health Research Division, Korea Institute of Oriental Medicine, Daejeon, Republic of Korea; ^2^KM Convergence Science, University of Science and Technology, Daejeon, Republic of Korea; ^3^Gwangju Alzheimer’s Disease and Related Dementias (GARD) Cohort Research Center, Chosun University, Gwangju, Republic of Korea; ^4^Department of Biomedical Science, Chosun University, Gwangju, Republic of Korea; ^5^Dementia Research Group, Korea Brain Research Institute, Daegu, Republic of Korea

**Keywords:** Alzheimer’s disease, mild cognitive impairment, event-related potential, electroencephalography, cognitive function, behavioral measure, screening tool

## Abstract

**Background:**

Alzheimer’s disease (AD) is among the leading contributors of dementia globally with approximately 60–70% of its cases. Current research is focused on the mild cognitive impairment (MCI), which is associated with cognitive decline but does not disrupt routine activities. Event-related potential (ERP) research is essential in screening patients with MCI. Low-density channel electroencephalography (EEG) is frequently used due to its convenience, portability, and affordability, making it suitable for resource-constrained environments. Despite extensive research on neural biomarkers for cognitive impairment, there is a considerable gap in understanding the effects on early stages of cognitive processes, particularly when combining physiological and cognitive markers using portable devices. The present study aimed to examine cognitive shortfalls and behavioral changes in patients with MCI using prefrontal selective attention ERP recorded from a prefrontal two-channel EEG device.

**Methods:**

We assessed cognitive decline using the Mini-Mental State Examination (MMSE) and the Seoul Neuropsychological Screening Battery (SNSB). We administered auditory selective attention tasks to 598 elderly participants, including those with MCI (160) and cognitively normal (CN) individuals (407). We conducted statistical analyses such as independent t-tests, Pearson’s correlations, and univariate and multiple logistic regression analyses to assess group differences and associations between neuropsychological tests, ERP measures, behavioral measures, and MCI prevalence.

**Results:**

Our findings revealed that patients with MCI demonstrated slower information-processing abilities, and exhibited poorer task execution, characterized by reduced accuracy, increased errors, and higher variability in response time, compared to CN adults. Multiple logistic regression analyses confirmed the association between some ERP and behavioral measures with MCI prevalence, independent of demographic and neuropsychological factors. A relationship was observed between neuropsychological scores, ERP, and behavioral measures.

**Discussion:**

The slower information processing abilities, and poor task execution in the MCI group compared to the CN individuals suggests flawed neurological changes and reduced attentional maintenance during cognitive processing, respectively. Hence, the utilization of portable EEG devices to capture prefrontal selective attention ERPs, in combination with behavioral assessments, holds promise for the identification of mild cognitive deficits and neural alterations in individuals with MCI. This approach could potentially augment the traditional neuropsychological tests during clinical screening for MCI.

## 1. Introduction

Dementia, with an estimated morbidity of 55 million and a yearly incidence of approximately 10 million is a prominent contributor of death and incapacity among the elderly population globally (WHO, 2022). The leading contributor of dementia is Alzheimer’s disease (AD), with approximately 60–70% of its cases (WHO, 2022). With an expected rise in the prevalence and related social costs of AD in the period between 2030 and 2050, current scientific and clinical research on AD prioritizes early detection of the intermediate stage between cognitively normal aging, mild cognitive impairment (MCI), and dementia (Dubois et al., 2007; Mantzavinos and Alexiou, 2017).

Mild cognitive impairment is a syndrome pronounced by cognitive decline which is higher than anticipated for a person’s age and level of education, without disrupting routine life activities (Gauthier et al., 2006). It could be an early indication of various degenerative, vascular, psychiatric, and medical disorders, with a potential to advance into degenerative conditions like AD dementia, frontotemporal dementia (FTD), and dementia with Lewy bodies (DLB). Furthermore, it might manifest as a symptom within non-degenerative conditions like vascular cognitive impairment (VCI), major depressive disorder, generalized anxiety disorders, uncompensated heart failure, and poorly managed diabetes mellitus (Petersen, 2016). Its further categorized into amnestic MCI (aMCI) if memory domain is affected or non-amnestic MCI (naMCI) if other cognitive domains are impaired. The quantity of impacted domains plays a crucial role in assessing the magnitude of underlying brain pathology, the disease’s impact, and the probability of transitioning to dementia. The yearly rate of progression from MCI to dementia fluctuates between 8 and 15% (Petersen, 2016) and its prevalence in persons ≥60 years is estimated to be between 15 and 20%, making it a rampant condition clinicians encounter (Gauthier et al., 2006).

This has attracted profound research interests as it’s crucial to promptly diagnose and treat individuals with a high risk of developing dementia prior to the emergence of substantial structural deficits. These individuals are suitable for therapeutic intervention (Missonnier et al., 2005). Furthermore, detecting individuals with increased risk of dementia is crucial in stopping disease progression, enabling the adoption of preventive healthcare, and easing potential emotional and financial pressures for both patients and caregivers. At present, patients with MCI and dementia are identified through assessment of cognitive function using neuropsychological tests. The Mini-Mental State Examination (MMSE) (Dick et al., 1984) is among the most extensively accessible and conveniently administered neuropsychological screening tests by primary care practitioners (Langa and Levine, 2014).

In South Korea, the Seoul Neuropsychological Screening Battery (SNSB) is a widely used comprehensive neuropsychological evaluation tool that provides scores in cognitive domains such as attention, memory, frontal/executive function, language, and visuospatial skills (Ryu and Yang, 2023). It provides key information for the evaluation of early cognitive decline, analysis of cognitive decline patterns, judgment of dementia severity, and differential diagnosis of dementia (Ryu and Yang, 2023). The complete administration of SNSB-II (the present version of SNSB) approximately takes 1 h and 45 min to 2 h. When exclusively conducting cognitive function tests, the duration is reduced to about 1 h to 1 h and 15 min (Ryu and Yang, 2023). This long duration renders the test impractical for patients with diminished attention spans and does not provide the global cognitive function (GCF) score, a valuable metric for continuous patient monitoring (Ahn et al., 2010).

Degenerative cognitive impairment is marked by a decline in several cognitive processes involving sensation, perception, cognition, and recognition, which precede higher-level cognitive functions (Perry and Hodges, 1999; Morrison et al., 2018). It’s often accompanied by the neurological alterations in the cerebral cortex and limbic system leading to deficits in learning, memory, language, and visuospatial skills (Corey-Bloom, 2002; Murman, 2015). While extensive research has been devoted to discovering the neural biomarkers responsible for cognitive impairment (Paitel et al., 2021), there is a considerable gap in understanding their effects on the early stages of cognitive processes, particularly when examining the combination of physiological and cognitive markers in a larger participant pool using a portable measurement device.

Using sensory or oddball event-related potential (ERP) paradigms, features that indicate impairments in cognitive processes have been studied (Nessler et al., 2007; Lai et al., 2010). Synchronized with an event, such as the start of a stimulus or the performance of a manual response (Kappenman and Luck, 2012), ERPs allow for the observation of a sequence of cognitive processes that unfold prior to the delivery of sensory information to the peripheral nervous system, persisting even after a behavioral response is executed (Woodman, 2010). In addition, they are more effective due to being readily accessible, cost-effectiveness, and high temporal specificity in contrast with other neuroimaging modalities (Paitel et al., 2021). The P300 ERP component signifies the cognitive processes associated with allocating attention and engaging working memory (Polich, 2007). It’s an expression of the central nervous system’s (CNS) activity involved in processing novel information while actively updating memory representations (Polich and Kok, 1995). Disparities in P300 observed during a simple stimulus discrimination task can reliably reflect individual variations in cognitive processing proficiency and swiftness (Polich and Kok, 1995), making it valuable for cognitive evaluation to identify and track the onset and progression of neurogenerative diseases (Medvidovic et al., 2013).

Recently, several studies have shown that EEG or ERP measures can be utilized to differentiate patients with MCI from cognitively normal persons or those with other cognitive impairments. For instance, Chapman et al. (2011) used the ERP obtained in the perceptual or cognitive paradigm to predict individuals with MCI who would later develop AD, using discriminant analysis with cross-validation accuracies of 70–78%. Ganapathi et al. (2022) obtained an area under the curve (AUC) of 0.72, differentiating between subjective cognitive impairment (SCI) and MCI. Bennys et al. (2007) observed significantly prolonged N200 and P300 latencies in patients with AD when compared to those with MCI or controls. Studies by Frodl et al. (2002) and Golob et al. (2002, 2007, 2009) provided more evidence suggesting a compromised P300 in individuals with MCI. However, some studies reported no differences in P300 measurements of amplitude (Papaliagkas et al., 2008; Lai et al., 2010; Cid-Fernández et al., 2014) and latency (Frodl et al., 2002; Papadaniil et al., 2016; Tsolaki et al., 2017; Cintra et al., 2018) between the CN and patients with MCI.

To enhance the early detection of MCI, there is a critical need for a diagnostic tool that is easily accessible, objective, and user-friendly, suitable for both clinical and non-clinical settings. Recent advancements in EEG technology have created the potential to develop a portable, cost-effective, and widely accessible EEG tool for MCI screening in primary care and outpatient settings (Doan et al., 2021; Smith, 2022). For instance, Khatun et al. (2019) achieved an accuracy of 87.9% in detecting MCI using a Support Vector Machine (SVM) with auditory ERPs obtained from a single-channel EEG device positioned at Fpz. Additionally, Choi et al. (2019) devised a regression model that exhibited a strong correlation of 0.757 in predicting MMSE scores in the elderly, utilizing resting-state prefrontal EEG data from a 2-channel EEG device (Fp1 and Fp2, per the 10-20 system). In a similar setup to Choi et al. (2019), Doan et al. (2021) achieved an Area Under the Receiver Operating Characteristic (AUROC) of 89.1% when distinguishing patients with Alzheimer’s Disease (AD) from healthy individuals, employing selective attention auditory ERPs.

This study aimed to assess the effectiveness of a portable EEG system in detecting MCI, with a specific focus on an auditory oddball task that elicits memory and attention ERPs, such as P300. By analyzing the ERP components related to selective attention, higher cognitive functions believed to be impaired in patients with MCI can be understood. We hypothesized that MCI-related neurological changes might impact ERP measures (Woodman, 2010), resulting in decreased task performance and ERP alterations in components associated with the oddball task in contrast to cognitively normal (CN) individuals of matching age. Furthermore, we anticipated a correlation between neuropsychological scores and both ERP and task-based behavioral measures.

## 2. Materials and methods

### 2.1. Participants

The present study included 598 participants, recruited between October 2019 and December 2020 at the Gwangju Alzheimer’s Disease and Related Dementia (GARD) center (Gwangju City, South Korea). We excluded 264 participants from the analysis because they were neither CN nor had MCI [*n* = 31], did not respond to target stimuli or had extreme errors compared to correct responses in the behavioral measures [*n* = 19], and had incomplete neuropsychological information [*n* = 2]. In addition, visual assessment was conducted by two experts, to identify a prominent P300 peak in the averaged ERPs. This criterion was used for participant inclusion, resulting in the exclusion of participants who did not display a discernible peak in the oddball ERP trace when compared to the standard ERP trace within the 300-600 ms time window [*n* = 212] (Supplementary Figure S2). This exclusion criterion was implemented in order to use the differential ERP method (Levi-Aharoni et al., 2020). This method is applicable when the data demonstrate two dependable time zero-crossing points, namely T1 and T2, between the oddball ERP and standard ERP.

The study participants were divided into two groups of similar ages: CN individuals and those diagnosed with MCI. This grouping was carried out as per the methodology described by Opwonya et al. (2022) which states, “All participants were examined through a clinical interview, which included assessment of the clinical dementia rating (CDR). The CN participants had a CDR score of 0. They had normal cognitive function with no evidence of brain atrophy, white matter changes, multiple lacunae, infarction, or other focal brain lesions on magnetic resonance imaging (MRI) scans. Participants with MCI met the Petersen criteria (Petersen, 2004) and had a CDR score of 0.5. Their neuropsychological test *z* scores were below −1.5 on at least one of five domain tests according to age, education, and sex-specific norms.”

The CN group had 239 participants (99 men and 140 women), with mean age ± standard deviation of 72.17 ± 5.72 years; the MCI group had 95 participants (42 men and 53 women), with mean age ± standard deviation of 74.13 ± 6.27 years (Table 1).

**Table 1:** Demographic characteristics and neuropsychological test domain scores.

Characteristic	CN, *N* = 239^1^	MCI, *N* = 95^1^	*T*-statistic	Value of *p*^2^
**Demographic characteristics**				
**Age**	72.17 (5.72)	74.13 (6.27)	−2.747	**0.006**
**Sex**			0.217	0.6
Female	140/239 (59%)	53/95 (56%)		
Male	99/239 (41%)	42/95 (44%)		
**EDUYR**	10.58 (4.37)	9.40 (4.78)	2.165	**0.031**
**Neuropsychological test domain scores**				
**MMSE**	27.62 (1.91)	26.04 (2.54)	6.192	**<0.001**
**Attention**	9.49 (2.21)	8.38 (1.90)	4.292	**<0.001**
**Language**	0.21 (0.25)	−0.13 (0.49)	8.251	**<0.001**
**Visuospatial**	0.52 (0.37)	0.00 (0.88)	7.541	**<0.001**
**Memory**	0.32 (0.59)	−0.55 (0.67)	11.621	**<0.001**
**Frontal**	0.22 (0.55)	−0.42 (0.71)	8.940	**<0.001**

^1^Mean (SD); n/N (%); ^2^Two Sample *t*-test; Pearson’s Chi-squared test; significant features (value of *p* ≤ 0.05) are bolded.

Every participant gave written informed consent, and the study received approval from the Institutional Review Board of Chonnam National University Hospital (IRB No. CNUH-2019-279).

### 2.2. Neuropsychological battery

In the present study, the latest version of the SNSB (SNSB II) was used to assess the cognitive function of the participants (Kang et al., 1997, 2003). Comprised of five cognitive domain scores—attention, language, memory, visuospatial, and frontal/executive functions—the SNSB II serves as a prominent neuropsychological screening battery in South Korea, usually employed to assess cognitive function in patients with MCI and dementia. We additionally employed the Korean Mini-Mental State Examination (K-MMSE) as the primary screening tool.

### 2.3. ERP recording

Event-related potentials were recorded using NeuroNicle FX2 (LAXTHA, Daejeon, South Korea) based on the 10-20 International system using 2 prefrontal monopolar scalp electrodes placed on Fp1 and Fp2 with a reference on the right earlobe. Additional details of our EEG/ERP experiments, as quoted below, were drawn from our earlier studies by Choi et al. (2019), Doan et al. (2021): “In addition, a bandstop filter was set between 55 and 65 Hz. All the EEG electrode contact impedances were maintained below 10 kΩ. The data were digitized in continuous recording mode at a sampling frequency of 250 Hz and 15-bit resolution. To eliminate muscle and eye movement artifacts and monitor sleepiness in the participants, qualified operators inspected the individuals and EEG traces during the recording. The operator guided the participants to remain comfortably seated with their eyes closed and alerted them whenever signs of behavioral or EEG drowsiness were detected. The EEG signals from the participants were acquired while they remained seated in an upright position under three sequential conditions: (1) spontaneous brain activity to establish background EEG signals in a resting state for 5 min (resting-state EEG), (2) sensory-evoked potentials for 8 min, and (3) a selective attention task to acquire the corresponding ERPs for 5 min. To elicit selective-attention ERP, we adopted an active auditory oddball task presenting 64 rare random target stimuli of 2,000 Hz (1/5 ratio) and 256 standard auditory stimuli of 750 Hz (4/5 ratio).”

In this study, only selective attentional ERPs were considered. Prior to the commencement of the experiment, all participants underwent evaluations of their auditory hearing acuity for both the rare tone (2,000 Hz) and the standard tone (750 Hz). Furthermore, participants were assessed for their capacity to distinguish between these tones (using earphones set at a uniform volume level of 70 dB). During the ERP experiment, participants were instructed to press a response key when they recognized the target stimuli. Recordings were made while participants kept their eyes closed in a soundproof room with regular illumination, ensuring a controlled environment for data collection.

### 2.4. Data pre-processing and feature extraction

The EEG data were analyzed using custom scripts written in *Python (version 3.8.16)*. The features extracted for the present study are described in Supplementary Table S1 and illustrated in Supplementary Figure S2.

#### 2.4.1. ERP measures

The EEG data for the two prefrontal channels (Fp1 and Fp2) were averaged to obtain EEG data from which subsequent pre-processing and feature extraction were performed. We extracted time epochs from −200 to 800 ms with respect to the presentation of stimuli from each of the correct trials (only the trials in which the standard stimuli were not responded to, and the target stimuli were responded to). The average standard and target ERPs were calculated by averaging the ERPs extracted from the EEG data for each participant’s stimuli. Each of the derived ERP traces (standard and target) was then baseline-corrected relative to a −200 to 0 ms period, and a moving average filter of order nine was applied to the final ERP traces. To isolate the ERP components, we derived the difference in ERP trace by subtracting the standard ERP trace from the target ERP trace, which was used to generate ERP variables (Levi-Aharoni et al., 2020) and 300-600 ms after stimulus onset was considered as the ERP time window.

The ERP measures extracted encompass various parameters, including Peak Amplitude (AMP), Latency (LAT), 50% Fractional Area Latency (FAL), onset zero-crossing point (T1), late zero-crossing point (T2), Area Under the Curve (AUC), the difference between T1 and T2 (T2T1), the difference between FAL and T1 (FALT1), and the difference between T2 and FAL (T2FAL).

#### 2.4.2. Behavioral measures

We also extracted features related to the behaviors of the participants during the ERP experiment. These include the number of incorrect or committed error responses (NI), error to correct ratio, i.e., ratio of all errors (incorrect and omitted error responses) to the correct responses (ER), response accuracy (ACC), weighted error percentile (WER), mean response time (RT), and variability in response time (RTSD), as measured by the standard deviation of the response times (Supplementary Table S1).

### 2.5. Statistical analysis

The statistical analyses were carried out using *R Studio (version 2022.07.2 + 576),* running on *R (version 4.1.3)* for Windows, including packages *gtsummary (version 1.6.1), ggplot2 (version 3.4.0)* and *corrplot (version 0.92)* (Wickham, 2016; Sjoberg et al., 2021; Wei and Simko, 2021; R Core Team, 2022) with a significance level of *α* = 0.05 for all tests. Independent sample t-tests were performed using Student’s t-test for continuous variables, and chi-squared tests were used for categorical variables. Univariate and multiple logistic regression analyses were performed to calculate the odds ratios associated with MCI for each ERP and behavioral measure while controlling for covariates such as age, sex, and years of education. The MMSE score was incorporated as an extra covariate to assess the independent relationship between ERPs, behavioral variables, and MCI. In addition, Pearson’s correlations were examined separately to understand the relationships between the neuropsychological domains and both attentional ERP and behavioral variables in the MCI and CN groups.

## 3. Results

### 3.1. Participant characteristics

The demographic information and neuropsychological characteristics of the participants considered for analysis in the present study are listed in Table 1.

The number of participants with MCI and CN was 95 and 239, respectively. The patients with MCI comprised 56% women and 44% men while the CN group comprised 59% women and 41% men. Patients with MCI were older than CN individuals, with mean age ± standard deviation of 74.13 ± 6.27 and 72.17 ± 5.72 years (*p* = 0.006) respectively. Furthermore, the patients with MCI had less years of education [9.40 ± 4.78] than CN individuals [10.58 ± 4.37] (*p* = 0.031). As expected, the patients with MCI had lower MMSE scores than CN individuals, with 26.04 ± 2.54 and 27.62 ± 1.91 score (*p* < 0.001) respectively. Overall, patients with MCI had lower MMSE scores and higher mean age than CN (Opwonya et al., 2022).

Patients with MCI had lower scores in all the SNSB II domains; attention [8.38 ± 1.90], language [−0.13 ± 0.49], visuospatial [0.00 ± 0.88], memory [−0.55 ± 0.67], and frontal [−0.42 ± 0.71] compared to CN individuals [9.49 ± 2.21, 0.21 ± 0.25, 0.52 ± 0.37, 0.32 ± 0.59, and 0.22 ± 0.55] (*p* < 0.001) respectively.

There were no statistically significant differences between the CN and MCI in sex.

### 3.2. ERP measures

**Figure 1:**
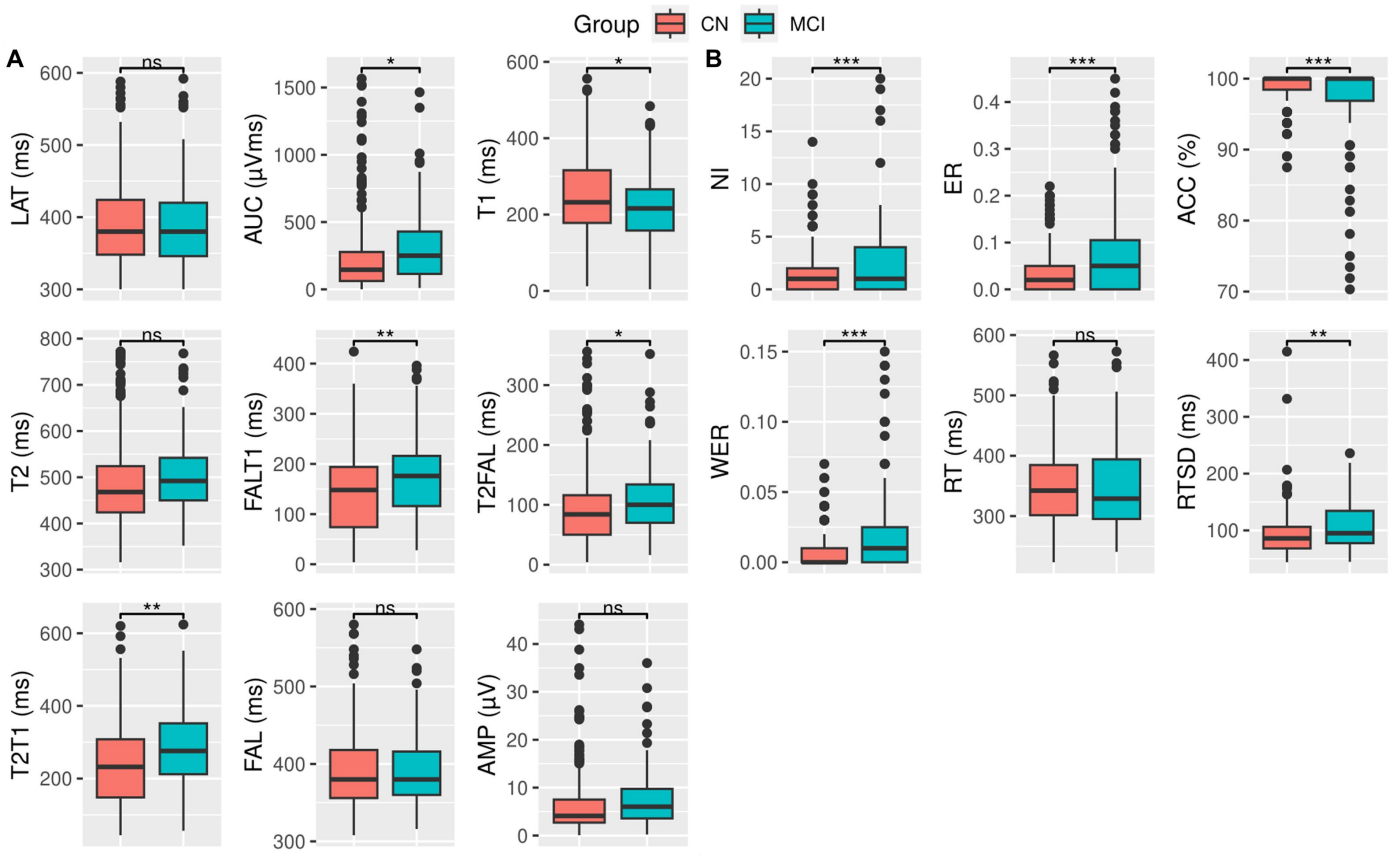
Box plot and *t*-test for **(A)** ERP variables and **(B)** behavioral measures for CN (red) and MCI (green) groups. Significance levels are denoted as follows: *** for *p* < 0.001, ** for *p* < 0.01, * for *p* ≤ 0.05, and ns for not significant. Detailed statistical scores and value of *p*s can be found in Table 2.

Patients with MCI showed a significantly larger AUC of the P300 duration [*t* = −2.13, *p* = 0.034] and an early onset zero-crossing time point (T1) [*t* = 2.38, *p* = 0.018] compared to the CN individuals, while exhibiting a higher difference between the onset zero-crossing time point and the 50% fractional area latency (FALT1) [*t* = −3.08, *p* = 0.002], the difference between the 50% fractional area latency and the late zero-crossing time point (T2FAL) [*t* = −2.25, *p* = 0.025], and the duration of the P300; the difference between the late and onset zero-crossing time points (T2T1) [*t* = −3.30, *p* = 0.001]. However, there were no significant differences in the distribution of peak amplitude (AMP), peak latency (LAT), late zero-crossing time point (T2), or 50% fractional area latency (FAL) among participants in either group (Figure 1A and Table 2).

**Table 2:** Participant’s ERP and behavioral measures.

**Characteristic**	**CN**, *N* = 239^1^	**MCI**, *N* = 95^1^	*T*-statistic	Value of *p*^2^
**ERP measures**				
**FAL**	390.88 (53.42)	392.21 (49.52)	−0.21	0.8
**AUC**	245.88 (297.27)	321.52 (281.66)	−2.13	**0.034**
**AMP**	6.58 (7.12)	7.99 (6.80)	−1.65	0.10
**LAT**	388.35 (62.41)	393.35 (63.52)	−0.66	0.5
**T1**	247.21 (101.51)	218.32 (96.81)	2.38	**0.018**
**T2**	484.75 (98.34)	503.66 (91.35)	−1.62	0.11
**FALT1**	143.67 (79.94)	173.89 (82.99)	−3.08	**0.002**
**T2FAL**	93.87 (65.18)	111.45 (62.10)	−2.25	**0.025**
**T2T1**	237.54 (119.41)	285.35 (119.62)	−3.30	**0.001**
**Behavioral measures**				
**NI**	1.52 (2.19)	2.71 (3.96)	−3.49	**<0.001**
**ER**	0.04 (0.05)	0.09 (0.12)	−5.82	**<0.001**
**ACC**	98.80 (2.23)	96.30 (7.02)	4.92	**<0.001**
**WER**	0.01 (0.01)	0.02 (0.04)	−5.47	**<0.001**
**RT**	347.80 (64.15)	348.80 (75.60)	−0.12	>0.9
**RTSD**	92.76 (39.96)	106.89 (40.72)	−2.90	**0.004**

^1^Mean (SD); ^2^Two Sample *t*-test; Significant variables (value of *p* ≤ 0.05) are bolded.

### 3.3. Behavioral measures

Compared to the CN individuals, patients with MCI exhibited significantly more incorrect responses (NI) [*t* = −3.49, *p* < 0.001], a higher ratio of error to correct responses (ER) [*t* = −5.82, *p* < 0.001], a greater response time variability (RTSD) [t = −2.90, *p* = 0.004], and higher weighted error percentile (WER) [*t* = −5.47, *p* < 0.001]. In addition, they showed a reduced response accuracy (ACC) [*t* = 4.92, *p* < 0.001].

However, the distribution of response time (RT) was similar between the two groups (Figure 1B and Table 2).

### 3.4. Logistic regression

Table 3 presents the odd ratios for ERP and behavioral measures for the risk of MCI.

**Table 3:** Estimated OR and 95% CI for ERP and behavioral measures derived from LR models.

	**Model 1**			**Model 2**			**Model 3**		
**Variables**	**OR**^**1**^	**95% CI**^**2**^	**Value of *p***	**OR**^**1**^	**95% CI**^**2**^	**Value of *p***	**OR**^**1**^	**95% CI**^**2**^	**Value of *p***
**ERP measures**									
**FAL**	1.03	0.81, 1.30	0.83	0.99	0.78, 1.26	0.97	0.98	0.75, 1.25	0.85
**AUC**	1.27	1.01, 1.59	**0.039**	1.25	0.99, 1.58	0.060	1.17	0.92, 1.49	0.20
**AMP**	1.21	0.96, 1.51	0.11	1.20	0.95, 1.52	0.12	1.14	0.89, 1.45	0.28
**LAT**	1.08	0.85, 1.37	0.51	1.06	0.83, 1.34	0.65	1.06	0.82, 1.36	0.66
**T1**	0.74	0.57, 0.95	**0.017**	0.74	0.57, 0.95	**0.019**	0.78	0.59, 1.01	0.059
**T2**	1.21	0.96, 1.53	0.11	1.16	0.91, 1.47	0.24	1.13	0.88, 1.45	0.33
**FALT1**	1.45	1.14, 1.85	**0.002**	1.42	1.11, 1.83	**0.005**	1.33	1.03, 1.73	**0.029**
**T2FAL**	1.29	1.03, 1.63	**0.028**	1.24	0.98, 1.57	0.071	1.22	0.95, 1.56	0.11
**T2T1**	1.48	1.17, 1.90	**0.001**	1.44	1.12, 1.85	**0.004**	1.36	1.05, 1.77	**0.019**
**Behavioral measures**									
**NI**	1.47	1.17, 1.90	**<0.001**	1.43	1.14, 1.85	**0.002**	1.42	1.12, 1.83	**0.004**
**ER**	1.92	1.49, 2.55	**<0.001**	1.83	1.42, 2.43	**<0.001**	1.72	1.33, 2.30	**<0.001**
**ACC**	0.57	0.42, 0.73	**<0.001**	0.59	0.43, 0.76	**<0.001**	0.63	0.46, 0.84	**0.001**
**WER**	1.87	1.45, 2.51	**<0.001**	1.80	1.39, 2.41	**<0.001**	1.69	1.28, 2.31	**<0.001**
**RT**	1.01	0.80, 1.28	0.90	1.00	0.78, 1.28	0.99	0.98	0.76, 1.27	0.90
**RTSD**	1.39	1.10, 1.79	**0.005**	1.31	1.03, 1.70	**0.027**	1.23	0.96, 1.59	0.10

^1^OR, odds ratio; ^2^CI, confidence interval; LR, logistic regression; Model 1: the unadjusted LR model; Model 2: LR model adjusted for demographic characteristics of age, sex and years of education; Model 3: LR model adjusted for demographic characteristics and the MMSE score. Significant variables (value of *p* ≤ 0.05) are bolded.

#### 3.4.1. ERP measures

In the unadjusted model, the odds ratios and corresponding 95% confidence intervals for the following variables were notably distinct from 1, indicating a potential association with the risk of MCI: AUC [OR = 1.27, *p* = 0.039], T1 [OR = 0.74, *p* = 0.017], FALT1 [OR = 1.45, *p* = 0.002], T2FAL [OR = 1.29, *p* = 0.026], and T2T1 [OR = 1.48, *p* = 0.001]. However, AMP, T2, LAT, and FAL did not show any significant risk of MCI.

After adjusting for the demographic characteristics of sex, age, and years of education, the second model showed that T1 [OR = 0.74, *p* = 0.019], FALT1 [OR = 1.42, *p* = 0.005], and T2T1 [OR = 1.44, *p* = 0.004] remained predictors for MCI. This confirmed their independence from demographic characteristics as predictors for MCI.

Further adjusting the second model with MMSE scores, FALT1 [OR = 1.33, *p* = 0.029] and T2T1 [OR = 1.36, *p* = 0.019] remained as predictors for MCI. An increase of 1 ms in the FALT1 and T2T1 levels increased the risk of MCI by 33 and 36%, respectively. This confirmed the true independence of FAT1 and T2T1 from both demographic characteristics and MMSE scores as predictors for MCI.

#### 3.4.2. Behavioral measures

The unadjusted model revealed that several behavioral measures had odds ratios and corresponding 95% confidence intervals notably distinct from 1, suggesting a risk of MCI. These measures included the NI [OR = 1.47, *p* < 0.001], ER [OR = 1.92, *p* < 0.001], ACC [OR = 0.57, *p* < 0.001], WER [OR = 1.87, *p* < 0.001], and RTSD [OR = 1.39, *p* = 0.005]. However, RT did not result in a significant risk for MCI.

After adjusting for the demographic characteristics of sex, age, and years of education, the second model showed that NI [OR = 1.43, *p* = 0.002], ER [OR = 1.83, *p* < 0.001], ACC [OR = 0.59, *p* < 0.001], WER [OR = 1.80, *p* < 0.001], and RTSD [OR = 1.31, *p* = 0.027] were predictors for MCI, confirming their independence from the influence of demographic characteristics.

Further adjusting the second model for MMSE score, revealed that NI [OR = 1.42, *p* = 0.004], ER [OR = 1.72, *p* < 0.001], ACC [OR = 0.63, *p* = 0.001], and WER [OR = 1.69, *p* < 0.001] persisted as predictors for MCI. Therefore, a unit increase in NI, ER, and WER increased the risk of MCI by 42, 72, and 69%, respectively. However, a unit decrease in the ACC increased the risk of MCI by 37%. However, RTSD is no longer considered a predictor for MCI.

### 3.5. Correlation

**Figure 2:**
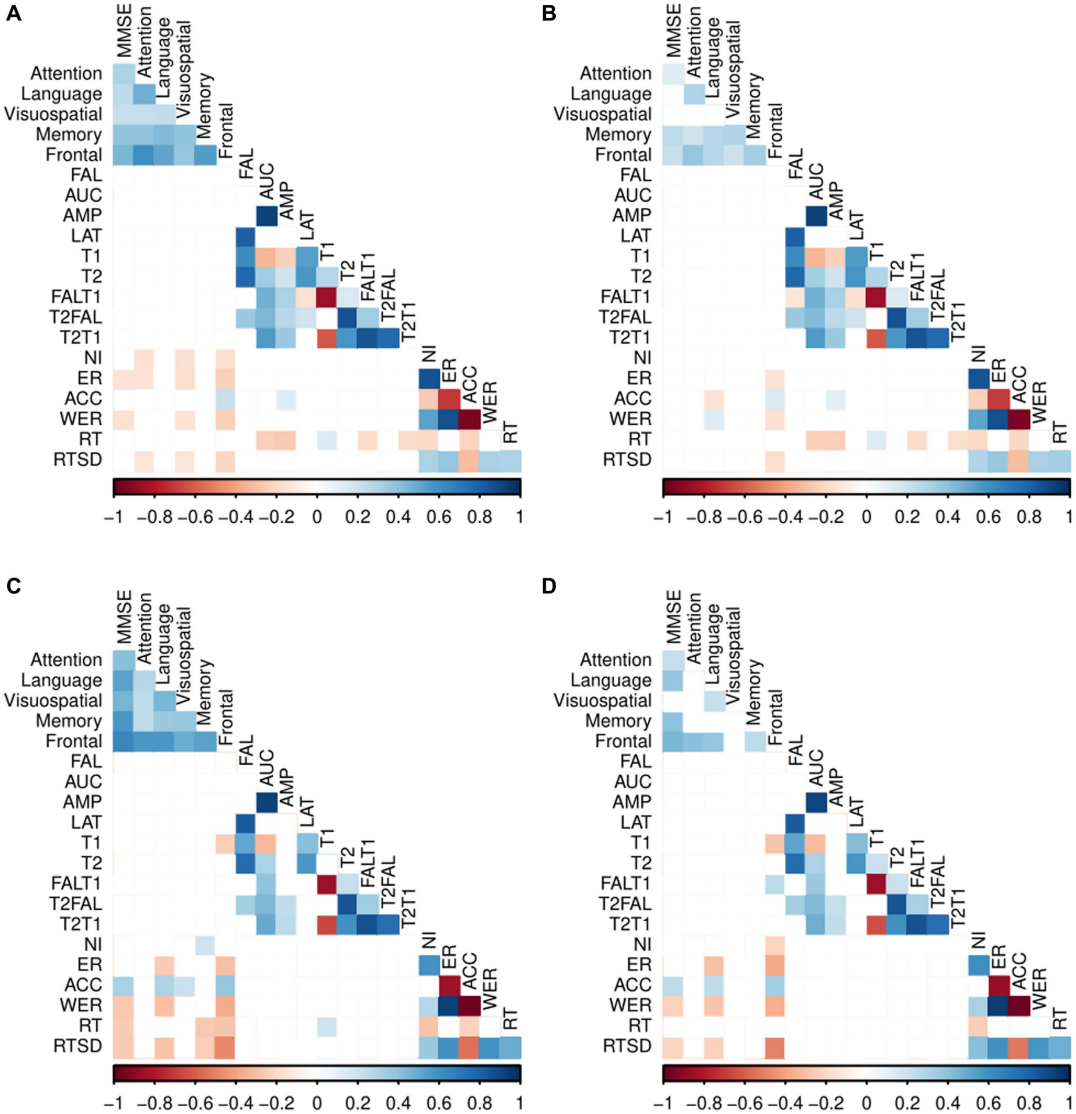
Pearson correlation coefficients between the two group’s ERP, behavioral measures, and neuropsychological measures. **(A)** Correlation between the ERP, behavioral measures, and neuropsychological tests in CN. **(B)** Partial correlations between the ERP, behavioral measures and neuropsychological tests in CN adjusted for age, sex and years of education. **(C)** Correlation between the ERP, behavioral measures, and neuropsychological tests in MCI. **(D)** Partial correlation between the ERP, behavioral measures and neuropsychological tests in MCI adjusted for age, sex and years of education. Exact partial correlation scores and value of *p*s are presented in Supplementary Tables S2, S3; Blank (white colored cells) represents no significant correlation between the variables.

To identify significant relationships between ERP, behavioral and neuropsychological measures (MMSE and SNSB II domain scores) in each participant group (CN and MCI), we calculated Pearson correlation coefficients while controlling for the effects of demographic characteristics of age, sex, and years of education (Figure 2).

#### 3.5.1. ERP measures and neuropsychological test scores

Among CN individuals, no significant correlations were observed between ERP variables and the neuropsychological tests.

For patients with MCI, we found a significant negative correlation between the frontal/executive function and T1 [*r* = −0.28, *p* = 0.01], and a positive correlation between the frontal and FALT1 [*r* = 0.26, *p* = 0.01]. However, no correlations were observed between the remaining ERP variables and neuropsychological tests.

#### 3.5.2. Behavioral measures and neuropsychological test scores

The CN participants displayed statistically significant negative correlations between frontal and various behavioral measures of task performance. Specifically, negative correlations were found between the frontal and the ER [*r* = −0.14, *p* = 0.03], WER [*r* = −0.16, *p* = 0.02], and RTSD [*r* = −0.18, *p* = 0.01], and between language and ACC [*r* = −0.14, *p* = 0.03].

However, there were significant positive correlations between the frontal and ACC [*r* = 0.17, *p* = 0.01] and between language and WER [*r* = 0.14, *p* = 0.03]. Notably, no significant correlations were found between behavioral measures and any of the MMSE, and the neuropsychological domains of attention, visuospatial function, and memory in CN individuals.

In patients with MCI, significant negative correlations were found between the MMSE and WER [*r* = −0.23, *p* = 0.03], MMSE and RTSD [*r* = −0.21, *p* = 0.04], language and ER [*r* = −0.30, *p* < 0.001], language and WER [*r* = −0.26, *p* = 0.01], language and RTSD [*r* = −0.22, *p* = 0.03], memory and RT [*r* = −0.21, *p* = 0.04], frontal and NI [*r* = −0.22, *p* = 0.03], frontal and ER [*r* = −0.37, *p* < 0.001], frontal and WER [*r* = −0.36, *p* < 0.001], and frontal and RTSD [*r* = −0.50, *p* < 0.001]. Furthermore, there were significant positive correlations between the MMSE and ACC [*r* = 0.25, *p* = 0.01], language and ACC [*r* = 0.26, *p* = 0.01], and frontal and ACC [*r* = 0.34, *p* < 0.001].

Behavioral measures showed no significant correlation with the neuropsychological domains of attention, visuospatial function, and memory in patients with MCI.

## 4. Discussion

This study examined the use of selective attention prefrontal ERP and task-related behavioral measures as possible biomarkers for identifying MCI using a portable EEG system. We analyzed the differences in ERP and task-related behavioral measures between individuals with MCI and CN individuals using an auditory oddball paradigm. Furthermore, we investigated the correlation between neuropsychological tests commonly used in MCI screening and both ERP and behavioral measures.

The ERP analysis indicated that patients with MCI displayed an elevated AUC and early T1, while demonstrating slower P300 timings of FALT1, T2FAL, and T2T1, compared to CN individuals. However, there were no notable differences in AMP, T2, LAT, or FAL between the two groups. After accounting for demographic factors of age, sex, and years of education, the T1, FALT1, and T2T1 ERP measures still showed a significant association with MCI. Even after additional consideration of the MMSE score, FALT1 and T2T1 retained their ability to differentiate between individuals with MCI and CN among the previously identified significant ERP variables. This suggests the true influence of ERP measures of FALT1 and T2T1 as possible predictors for MCI, independent of demographic characteristics and neuropsychological tests.

Specifically, we did not observe any significant difference in the amplitude between the two groups, although the MCI group had larger amplitudes than the CN group. This was similar with prior studies (Papaliagkas et al., 2008; Lai et al., 2010; Cid-Fernández et al., 2014) that reported no difference in P300 amplitudes between patients with MCI and CN individuals. These results indicate that both groups comparably mobilized the attentional resources needed for stimuli categorization and updating the context in working memory (Gironell et al., 2005). These results could be attributed to several factors. First, it is possible that patients with MCI compensate for cognitive deficits by recruiting additional neural resources (Scheller et al., 2014), leading to increased mobilization of attentional resources needed for stimulus categorization and context updating in working memory; (Gironell et al., 2005) thus, demonstrating analogous performance to CN individuals. Second, differences in the ERP tasks used or variations among studies that employed similar auditory oddball tasks and discrepancies in the inclusion criteria for the MCI group [owing to the heterogeneity of the MCI patients (Petersen, 2004; Delano-Wood et al., 2009)] may have contributed to the discrepancies in the results of most of the other studies.

Next, we did not observe any statistically significant differences in the P300 latency measures of LAT and FAL between MCI and CN groups. This result suggests that the cognitive decline observed in our MCI group did not influence the duration required for the assessment and categorization of auditory target stimuli within working memory. This agrees with prior studies (Frodl et al., 2002; Papadaniil et al., 2016; Tsolaki et al., 2017; Cintra et al., 2018) that used the auditory oddball task and found no significant differences in the latency between the MCI and CN groups. Evidence has shown that the P300 peak latencies are more accurate in the prodromal phase when patients are typically younger than 70 years (Bennys et al., 2007). This could be a reason for our findings, as the participants in the present study were generally older (mean age, 73.15 years) and it’s possible that the increased neural degeneration associated with aging could render oddball tasks excessively demanding on cognitive resources, potentially making it challenging to attain a consistent distinction between patients and cognitively normal individuals (Howe et al., 2014).

In contrast to the behavior of P300 amplitude or latencies, in the novel difference measures, we observed that patients with MCI had lengthened P300 timings for T2T1, FALT1 and T2FAL, and a shorter T1 duration compared to the CN individuals. T2T1, the difference in the zero-crossing time points of the P300 component suggests its duration and can be an index of cognitive processing time. The prolonged FALT1, T2FAL, and T2T1 in patients with MCI compared to the CN individuals indicated that the CN group possessed faster information processing and decision-making abilities than the MCI group. This delay within the MCI group during the task implies a need for extra time to process information, hinting at a possible impairment in cognitive ability (van Deursen et al., 2009) and a possible neocortical dysfunction, which predicts further cognitive decline (Lai et al., 2010). T1 represents the time point extracted from the isolated P300 component using a differential wave approach (Vogel et al., 1998; Luck et al., 2009) where the P300 trace deviates from the baseline (Kiesel et al., 2008). Short T1 duration suggests an early onset of P300, indicating that information processing may commence earlier in the MCI group than in the CN group. Once adjustments were made for demographic measures of sex, age, and education level, T1, FALT1, and T2T1 continued to exhibit noteworthy associations as predictors for MCI. Despite further adjustment for MMSE score, FALT1 and T2T1 remained significant predictors for MCI. This suggests that FALT1 and T2T1 are independent features for MCI screening and can be used in place or as supplements to the MMSE score.

Similarly, we found a significantly larger AUC in patients with MCI than in CN individuals. The AUC quantifies the overall pattern of the P300 waveform, providing insights into the level of cognitive processing across a temporal span (Kim et al., 2013). Reiterating this understanding with respect to the AUC results, suggests that patients with MCI perform more processing and use more effort and attentional resources to complete the same task than CN individuals. MCI refers to a state of cognitive impairment, primarily impacting memory and other cognitive domains, and an increased ERP AUC in patients with MCI compared to healthy individuals could also reflect altered cognitive processing. This suggests that patients with MCI may compensate for cognitive deficits by recruiting additional neural resources or exhibiting hyperactivation in certain brain regions (Scheller et al., 2014). Nonetheless, upon accounting for demographic measures of age, sex, and education level, the significance of the AUC diminished, suggesting that these factors might have exerted considerable influence on the extent of cognitive processing over time within the MCI group.

In the analysis of task-related behavioral measures, patients with MCI demonstrated significantly increased RTSD, NI, ER, and WER compared to CN individuals. Additionally, the MCI group showed reduced accuracy (ACC) in the task. The elevated RTSD in MCI patients indicates an underlying functional integrity that could potentially serve as a differentiator between MCI and CN individuals, suggesting RTSD’s sensitivity to cognitive decline, pathological load, and neurological dysfunction (Strauss et al., 2007; McLaughlin et al., 2010). It is likely that RTSD might be more pronounced in the presence of early stage and advanced dementia, further supported by previous studies that investigated response time variability in MCI or AD (Gorus et al., 2008; Burton et al., 2009; Bielak et al., 2010; Phillips et al., 2013). Furthermore, the increased error-related measures (NI, ER, and WER) in MCI patients suggest a decline in the capacity to sustain attention and manage actions while engaging in cognitive task processing (Vecchio and Määttä, 2011). This could imply impairments in the brain’s ability to filter irrelevant information and allocate attention efficiently to relevant stimuli, leading to heightened distractibility and difficulty in accurately identifying target stimuli in an ERP task, resulting in more incorrect responses (Lorenzo-López et al., 2016). These findings are consistent with prior studies that reported a higher frequency of errors in MCI patients compared to CN individuals (Cid-Fernández et al., 2014; Zurrón et al., 2018). Notably, the statistical significance of RTSD, NI, ER, WER, and ACC as predictors for MCI remained intact, even after adjusting for age, sex, and education level, indicating their robust predictive power independent of these demographic factors. Additionally, after further adjustment for the MMSE score, the significance of NI, ER, WER, and ACC as predictors for MCI persisted, underscoring their true influence as independent predictors for MCI.

This study also investigated the correlation between neuropsychological measures and both ERP and behavioral measures. We controlled for demographic characteristics to ensure that any correlations observed between neuropsychological measures and both ERP and behavioral measures were not confounded by demographic factors of age, sex, and years of education. Certain correlations seemed to be linked to demographic factors, as their impact diminished upon controlling for these factors. In the MCI group, we found negative and positive correlations between the frontal function and the T1 and FALT1, respectively (Figure 2).

We observed mild-to-moderate correlations between the behavioral measurements and neuropsychological scores. Particularly, we found significant negative correlations between the NI and frontal/executive function in patients with MCI but not in the CN individuals. Furthermore, we observed polarized correlations within the language domain when analyzing both ACC and WER in both groups. In the CN group, we found negative correlations between language and ACC, while in the MCI group, we observed the opposite, with language showing a positive correlation with ACC. In contrast, the CN group displayed positive correlations between language and WER, whereas the MCI group demonstrated the reverse pattern. Moreover, we found significant negative correlations between the frontal and both the ER and WER in both groups. This implies that more errors during a task could indicate reduced executive function, which is a manifestation of age-related cognitive decline. This suggests that neurodegeneration taking place in the brain regions responsible for advanced cognitive functions and task execution advances laterally (Opwonya et al., 2022). Additionally, negative correlations between RTSD and frontal domain scores were observed in both groups. In addition, there were significant negative correlations between MMSE and RTSD and between language and both the ER and RTSD in the MCI group but not in the CN group. This implies that the higher the RTSD, the lower the MMSE and language function scores. These negative correlations suggest a link between onset cognitive decline and lapses in attention (Datta et al., 2007). Lastly, there was a significant positive correlation between the frontal and the ACC in both groups. This indicates that the higher the accuracy, the greater the executive function performance.

The ERP and behavioral measures capable of discriminating MCI independently from neuropsychological screening tests such as the MMSE will be good replacements or complements for the MMSE, owing to certain constraints of screening tools like the MMSE which include limitations stemming from language or educational differences, the potential for a learning effect, and reduced sensitivity in the early stages of cognitive decline (Scazufca et al., 2009; Carnero-Pardo, 2014). These studies (Chapman et al., 2007, 2011; Cecchi et al., 2015; Stuckenschneider et al., 2020; Doan et al., 2021; Ganapathi et al., 2022) developed diagnostic systems based on EEG/ERP measurements, so some of the relevant features identified in our work can be used to improve MCI or early AD screening models.

This study had several limitations. First, the generalizability of our findings may be limited, as we examined ERP measures only in ethnically Korean participants. Second, the MCI participants were not categorized into amnestic or non-amnestic phenotypes because of their smaller number compared to the healthy participants. This heterogeneity of patients with MCI may have contributed to discrepancies in the results (Petersen, 2004; Delano-Wood et al., 2009). Third, we deployed a rigorous exclusion criterion by eliminating participants who did not have a P300 ERP component onset or late zero-crossing points. This methodological drawback resulted in the exclusion of a significant number of participants. Fourth, because our results were based on a single EEG recording, there’s a potential for the cognitive function of patients with MCI to evolve over time, which could involve either a return to normal function or progression to other conditions. Thus, further investigation into the longitudinal changes of ERP measures is desirable to validate our results. It is also necessary to conduct prospective studies aimed at establishing the clinical implications and significance of the ERP measures utilized in the current study.

In conclusion, our study aimed to demonstrate the potential of prefrontal ERP measures from a portable EEG device for distinguishing patients with MCI from CN individuals. We provided a comprehensive description of these ERP measures and examined their relationships with neuropsychological tests commonly used in MCI screening. Our findings showed that patients with MCI demonstrated slower information processing abilities, initiated information processing earlier and exhibited poor task execution than CN. Logistic regression analysis for MCI prediction showed that some ERP and behavioral measures remained statistically significant even after adjusting for demographic characteristics and neuropsychological test scores, providing further evidence that ERP and behavioral measures could serve as valuable complements to neuropsychological tests for screening mild cognitive deficits. In future studies, there are possible areas to explore. First, it is important to validate our findings by broadening the study to encompass a more diverse ethnic population. Furthermore, there is need to establish links between the identified ERP measures and neurodegeneration biomarkers, as well as functional or structural neuroimaging data. Moreover, in the pursuit of enhancing predictive models for MCI, inclusion of these ERP measures, either independently or in combination with other non-invasive techniques like eye-tracking measurements could be considered.

## Data availability statement

The raw data supporting the conclusions of this article will be made available by the authors, without undue reservation.

## Ethics statement

The studies involving humans were approved by Institutional Review Board of Chonnam National University Hospital. The studies were conducted in accordance with the local legislation and institutional requirements. The participants provided their written informed consent to participate in this study.

## Author contributions

JE: Conceptualization, Data curation, Formal analysis, Investigation, Software, Validation, Writing – original draft, Writing – review & editing, Methodology. WK: Data curation, Formal analysis, Writing – review & editing, Methodology, Writing – original draft. KK: Data curation, Methodology, Project administration, Writing – review & editing. KL: Methodology, Writing – review & editing, Data curation. JK: Conceptualization, Formal analysis, Funding acquisition, Investigation, Methodology, Project administration, Software, Supervision, Validation, Writing – original draft, Writing – review & editing, Data curation.
