# Corrigendum: Prefrontal event-related potential markers in association with mild cognitive impairment

**DOI:** 10.3389/fnagi.2023.1333781

**Published:** 2023-11-22

**Authors:** Joel Eyamu, Wuon-Shik Kim, Kahye Kim, Kun Ho Lee, Jaeuk U. Kim

**Affiliations:** ^1^Digital Health Research Division, Korea Institute of Oriental Medicine, Daejeon, Republic of Korea; ^2^KM Convergence Science, University of Science and Technology, Daejeon, Republic of Korea; ^3^Gwangju Alzheimer's Disease and Related Dementias (GARD) Cohort Research Center, Chosun University, Gwangju, Republic of Korea; ^4^Department of Biomedical Science, Chosun University, Gwangju, Republic of Korea; ^5^Dementia Research Group, Korea Brain Research Institute, Daegu, Republic of Korea

**Keywords:** Alzheimer's disease, mild cognitive impairment, event-related potential, electroencephalography, cognitive function, behavioral measure, screening tool

In the published article, there was an error. The results of the ERP timings' differences between patients with MCI and healthy individuals were misreported, as “lower” or “reduced” instead of “higher” or “increased/slowed.”

A correction has been made to **Results**, *ERP measures*, paragraph 1. This sentence previously stated:

“Patients with MCI showed a significantly larger AUC of the P300 duration [*t* = −2.13, *p* = 0.034] and an early onset zero-crossing time point (T1) [*t* = 2.38, *p* = 0.018] compared to the CN individuals, while exhibiting a lower difference between the onset zero-crossing time point and the 50% fractional area latency (FALT1) [*t* = −3.08, *p* = 0.002], the difference between the 50% fractional area latency and the late zero-crossing time point (T2FAL) [*t* = −2.25, *p* = 0.025], and the duration of the P300; the difference between the late and onset zero-crossing time points (T2T1) [*t* = −3.30, *p* = 0.001].”

The corrected sentence appears below:

“Patients with MCI showed a significantly larger AUC of the P300 duration [*t* = −2.13, *p* = 0.034] and an early onset zero-crossing time point (T1) [*t* = 2.38, *p* = 0.018] compared to the CN individuals, while exhibiting a **higher** difference between the onset zero-crossing time point and the 50% fractional area latency (FALT1) [*t* = −3.08, *p* = 0.002], the difference between the 50% fractional area latency and the late zero-crossing time point (T2FAL) [*t* = −2.25, *p* = 0.025], and the duration of the P300; the difference between the late and onset zero-crossing time points (T2T1) [*t* = −3.30, *p* = 0.001].”

A correction has also been made to **Discussion**, paragraph 2. This sentence previously stated:

“The ERP analysis indicated that patients with MCI displayed an elevated AUC and early T1, while demonstrating reduced P300 timings of FALT1, T2FAL, and T2T1, compared to CN individuals.”

The corrected sentence appears below:

“The ERP analysis indicated that patients with MCI displayed an elevated AUC and early T1, while demonstrating **slower** P300 timings of FALT1, T2FAL, and T2T1, compared to CN individuals.”

The authors apologize for this error and state that this does not change the scientific conclusions of the article in any way. The original article has been updated.

