# Supplementary material for: Prefrontal event-related potential markers in association with mild cognitive impairment
Source: Front Aging Neurosci. 2023 Oct 19;15:1273008. doi: 10.3389/fnagi.2023.1273008 (PMC10620700; doi:10.3389/fnagi.2023.1273008)
Supplement: Supplementary file 1 [file Data_Sheet_1.PDF]

Table S1: ERP and behavioral measures

Variable	Unit	Description	Effect on cognitively Impaired patients
**ERP Measures**			
AMP	µV	**Peak Amplitude**: The largest point (peak) in the measurement window surrounded by lower voltages on both sides (Luck, 2014).	Patients with AD dementia showed lower amplitude for ERP (Golob et al., 2002) (Cecchi et al., 2015)
LAT	ms	**Latency**: The timepoint corresponding to the peak amplitude starting from the stimulus onset.	Increased latency was reported in AD (Golob et al., 2007)(Doan et al., 2021) and MCI (Zurrón et al., 2018) patients
T1	ms	The onset zero-crossing timepoint of the P300 component.	-
T2	ms	The late zero-crossing timepoint of the P300 component.	-
AUC	µVms	**P300 duration area under the curve**: The sum of the amplitudes above the zero ERP activity in the T1 to T2 ms time window after the auditory stimulus onset that measures the duration of the P300 component.	-
FAL	ms	**Fractional area Latency:** The timepoint in which the area in the 300 to 600 ms window is divided into two equal parts. As demonstrated by (Luck, 2014) , it is less sensitive to noise compared to the peak latency and tended to be the most reliable way of measuring changes in latency across conditions or groups(Andrea Kiesel, Jeff Miller, Pierre Jolicœur, 2007).	Increased latency was reported in AD (Golob et al., 2007)(Doan et al., 2021) and MCI (Zurrón et al., 2018) patients.
FALT1	ms	Difference between the 50% fractional area latency and the onset zero-crossing timepoint in the ERP trace. (FALT1 = FAL-T1).	-

Table S1: ERP and behavioral measures

Variable	Unit	Description	Effect on cognitively Impaired patients
T2FAL	ms	Difference between the late zero-crossing timepoint and the 50% fractional area latency for the ERP trace. (T2FAL = T2-FAL).	-
T2T1	ms	Difference between T2 and T1 for the ERP trace. (T2T1 = T2-T1).	-
**Behavioral Measures**			
NI	-	Number of incorrect responses (commission errors) during the ERP experiment.	Reduced accuracy in ERP-related tasks in dementia patients (Cecchi et al., 2015).
ER	-	Ratio of all the errors (omissions and incorrect or commissions) to correct responses; ER = (NO + NI)/NC<br>Where; NC = Number of correct responses<br>NO = Number of omitted responses	Reduced accuracy in ERP-related tasks in dementia patients (Cecchi et al., 2015).
ACC		**Percentage of correct responses**;<br>ACC= (NC/64) *100	Reduced accuracy in ERP-related tasks in dementia patients (Cecchi et al., 2015).<br>Reduced number of correct responses reported in ERP-related tasks in dementia patients (Doan et al., 2021).
WER	-	**Weighted Error Percentile**;<br>WER = NI + 4 x (64- NC)/ (256 + 64 x 4)<br>No. of target(background) stimuli = 64(256)<br>4 is the weight balancing factor (Background/No. of Target) = (256/64)	Increased weighted error percentile was reported in dementia patients (Doan et al., 2021).

Table S1: ERP and behavioral measures

Variable	Unit	Description	Effect on cognitively Impaired patients
RT	ms	The average of the response times (time between target stimuli onset and a button press) during the experiment.	Reduced response time in target ERP detection-related tasks in dementia patients (Cecchi et al., 2015).<br>MCI patients showed longer response times (Golob et al., 2007) (Chen et al., 2021) (Ellen Gorus, Rudi De Raedt, Margareta Lambert, Jean-Claude Lemper, Tony Mets, 2008).
RTSD	ms	The standard deviation of the response times; a measure of the variability of the response times during the ERP experiment.	Persons with cognitive deterioration demonstrated more intra-individual variability in response time than cognitively healthy elderly (Christ et al., 2018; Ellen Gorus, Rudi De Raedt, Margareta Lambert, Jean-Claude Lemper, Tony Mets, 2008; Phillips et al., 2013).

Table S2: Partial Correlations between ERP measures and neuropsychological tests in the two groups

		MMSE	SNSB Domains				
	ERP	MMSE	Attention	Language	Visuospatial	Memory	Frontal
CN	AUC	-0.10 (0.11)	0.00 (0.98)	0.02 (0.81)	0.09 (0.15)	0.04 (0.51)	-0.08 (0.23)
	AMP	-0.08 (0.21)	-0.02 (0.76)	-0.03 (0.65)	0.04 (0.51)	0.04 (0.45)	-0.08 (0.24)
	LAT	-0.01 (0.85)	0.00 (0.95)	-0.01 (0.91)	-0.05 (0.43)	0.00 (0.95)	0.01 (0.91)
	FAL	0.00 (0.95)	0.10 (0.14)	-0.05 (0.41)	-0.01 (0.88)	0.02 (0.76)	0.08 (0.24)
	T1	0.09 (0.16)	0.05 (0.42)	-0.03 (0.65)	-0.03 (0.63)	0.03 (0.63)	0.10 (0.12)
	T2	0.01 (0.89)	0.11 (0.09)	0.00 (0.98)	0.01 (0.87)	0.02 (0.72)	0.05 (0.44)
	FALT1	-0.11 (0.08)	0.00 (0.98)	0.00 (0.98)	0.03 (0.62)	-0.03 (0.69)	-0.08 (0.23)
	T2FAL	0.01 (0.87)	0.08 (0.19)	0.04 (0.52)	0.02 (0.71)	0.02 (0.77)	0.01 (0.84)
	T2T1	-0.07 (0.28)	0.04 (0.49)	0.02 (0.71)	0.03 (0.59)	-0.01 (0.91)	-0.05 (0.48)
MCI	AUC	-0.09 (0.37)	0.04 (0.71)	-0.13 (0.21)	-0.01 (0.95)	0.07 (0.50)	0.08 (0.45)
	AMP	-0.08 (0.44)	0.12 (0.28)	-0.10 (0.33)	0.00 (0.98)	0.08 (0.44)	0.09 (0.40)
	LAT	-0.03 (0.76)	-0.08 (0.45)	-0.02 (0.85)	-0.05 (0.63)	0.05 (0.61)	-0.03 (0.80)
	FAL	-0.13 (0.19)	-0.05 (0.61)	-0.05 (0.54)	-0.04 (0.73)	-0.06 (0.55)	-0.11 (0.27)
	T1	-0.02 (0.86)	-0.11 (0.33)	-0.06 (0.58)	0.04 (0.69)	-0.08 (0.46)	**-0.28 (0.01)**
	T2	-0.14 (0.19)	-0.08 (0.43)	-0.07 (0.48)	-0.01 (0.91)	-0.01 (0.94)	-0.06 (0.56)
	FALT1	-0.06 (0.55)	0.09 (0.40)	0.04 (0.70)	-0.07 (0.49)	0.05 (0.61)	**0.26 (0.01)**
	T2FAL	-0.09 (0.37)	-0.08 (0.45)	-0.06 (0.54)	0.01 (0.91)	0.04 (0.72)	0.00 (0.98)
	T2T1	-0.09 (0.38)	0.02 (0.86)	-0.01 (0.95)	-0.04 (0.68)	0.06 (0.59)	0.18 (0.08)

Table S2: Pearson correlation coefficient(p-value); CN: cognitively normal; MCI: Mild cognitive impairment; significant correlations (p-values ≤ 0.05) are bolded; Adjusted for age, sex and education.

Table S3: Partial correlation coefficients between behavioral measures and neuropsychological test scores

		MMSE	SNSB Domains				
	ERP	MMSE	Attention	Language	Visuospatial	Memory	Frontal
CN	NI	-0.06 (0.32)	-0.09 (0.17)	0.05 (0.46)	-0.12 (0.07)	0.00 (0.99)	-0.06 (0.33)
	ER	-0.08 (0.24)	-0.06 (0.40)	0.10 (0.11)	-0.12 (0.07)	-0.02 (0.76)	**-0.14 (0.03)**
	ACC	0.05 (0.41)	-0.03 (0.68)	**-0.14 (0.03)**	0.05 (0.40)	0.03 (0.62)	**0.17 (0.01)**
	WER	-0.08 (0.20)	-0.01 (0.83)	**0.14 (0.03)**	-0.09 (0.18)	-0.04 (0.55)	**-0.16 (0.02)**
	RT	0.06 (0.34)	-0.02 (0.81)	-0.03 (0.66)	-0.02 (0.72)	0.02 (0.80)	-0.11 (0.10)
	RTSD	-0.01 (0.87)	-0.08 (0.22)	0.09 (0.16)	-0.10 (0.11)	0.08 (0.24)	**-0.18 (0.01)**
MCI	NI	0.02 (0.87)	-0.16 (0.11)	-0.19 (0.07)	0.00 (0.99)	0.12 (0.24)	**-0.22 (0.03)**
	ER	-0.20 (0.06)	-0.14 (0.17)	**-0.30 (<0.001)**	-0.09 (0.38)	0.04 (0.72)	**-0.37 (<0.001)**
	ACC	**0.25 (0.01)**	0.07 (0.51)	**0.26 (0.01)**	0.10 (0.34)	0.03 (0.75)	**0.34(<0.001)**
	WER	**-0.23 (0.03)**	-0.09 (0.36)	**-0.28 (0.01)**	-0.09 (0.40)	-0.01 (0.93)	**-0.36 (<0.001)**
	RT	-0.14 (0.17)	-0.01 (0.90)	-0.02 (0.84)	0.14 (0.18)	-0.19 (0.07)	-0.19 (0.07)
	RTSD	**-0.21 (0.04)**	-0.12 (0.23)	**-0.22 (0.03)**	0.02 (0.82)	-0.17 (0.09)	**-0.50 (<0.001)**

Table S3: Pearson correlation coefficient(p-value); CN: cognitively normal; MCI: Mild cognitive impairment; significant correlations (p-values ≤ 0.05) are bolded; Adjusted for age, sex and education.

Figure S1. Composition of the participants in the study.

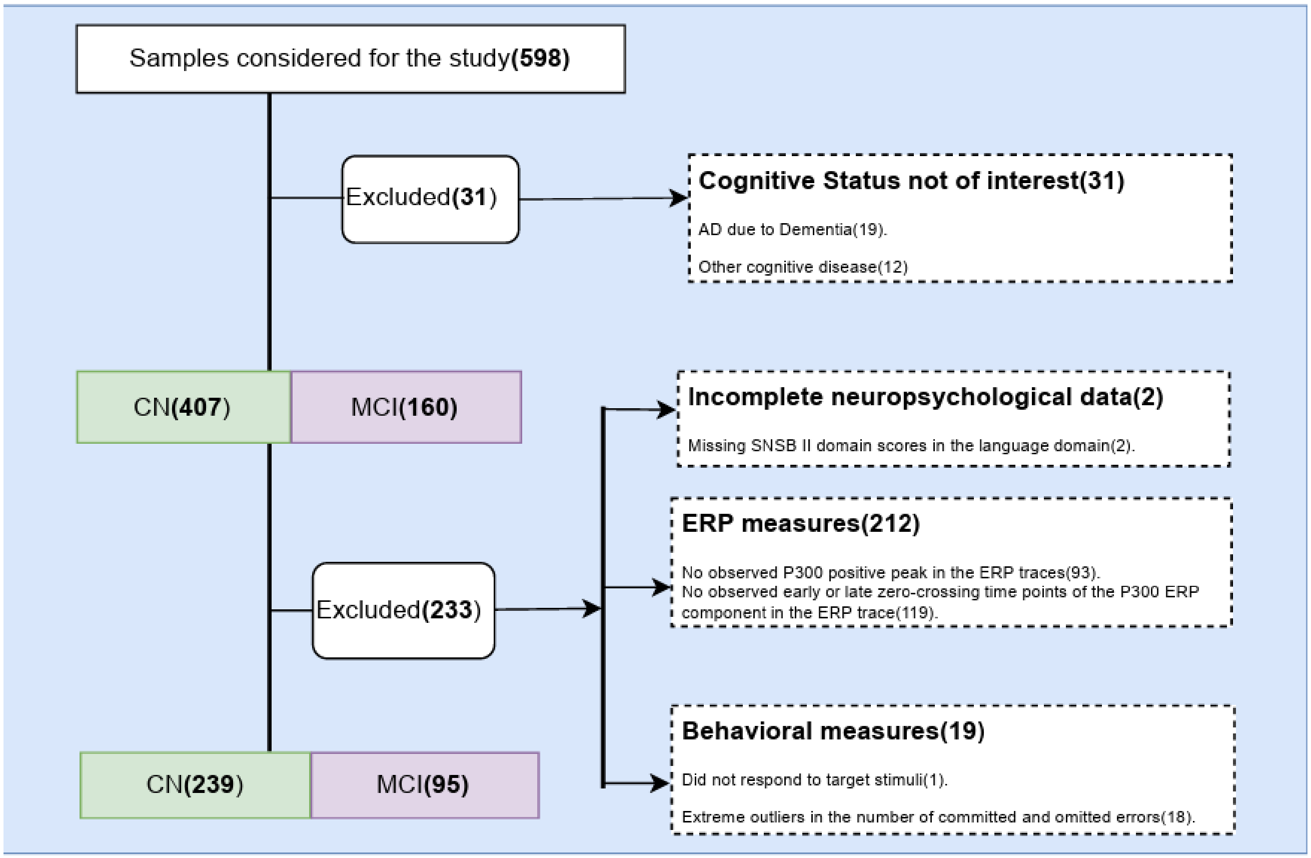

Figure S1. Summary of the composition of the participants in the study; CN: Cognitively normal; MCI: Mild cognitive impairment; AD: Alzheimer's disease; Values are in parentheses.

Figure S2. ERP measures considered in the study.

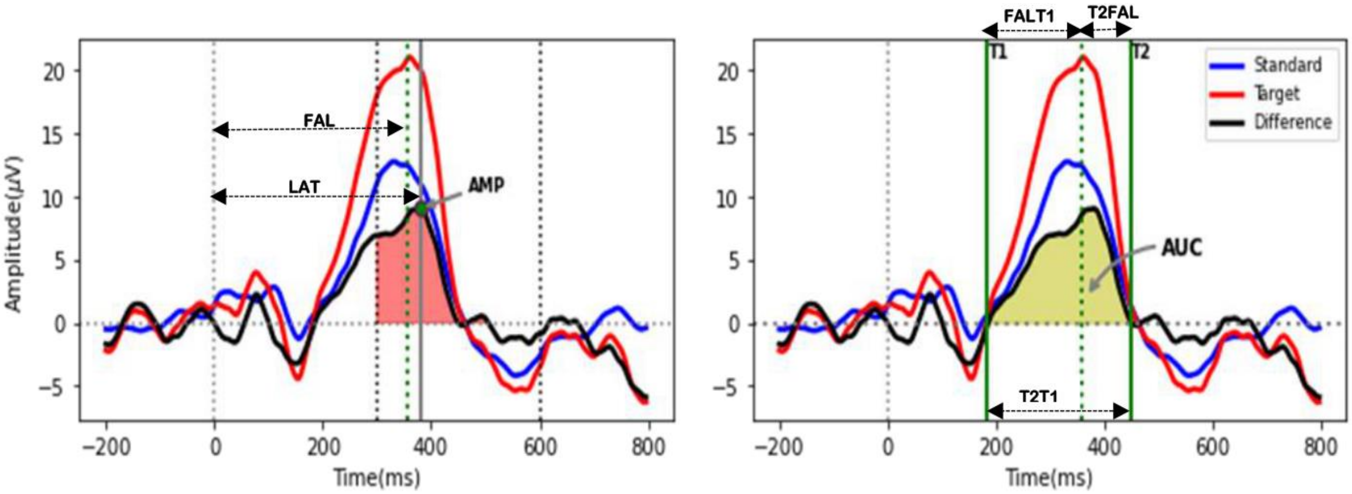

Figure S2: Event-related potentials averaged over all target (red) and standard (blue) trials for the averaged Fp1 and Fp2 electrodes brain activity are shown for a representative subject. The difference between the target and standard traces (solid black) were used to determine the ERP measures for each subject; ***Left***: Peak amplitude - **AMP**: the maximum voltage of the ERP trace; Latency - **LAT**: time between the stimulus onset and time corresponding to AMP; 50% fractional area latency - **FAL** (dotted vertical green line): timepoint that equally divides the area (shaded in red) of the 300 to 600 ms time window; ***Right***: **T1** and **T2** (solid green lines) : onset and late zero-crossing points of the difference trace around the FAL; P300 duration area under the curve - AUC (shaded yellow area): the area between T1 and T2 window; **T2T1** : the difference between T1 and T2; **FALT1**: the difference between FAL and T1; **T2FAL**: the difference between T2 and FAL (see Table S1 for descriptions of these variables)

References

Andrea Kiesel, Jeff Miller, Pierre Jolicœur, B. B. (2007). Measurement of ERP latency differences: A comparison of single-participant and jackknife-based scoring methods. *Psychophysiology*.

Cecchi, M., Moore, D. K., Sadowsky, C. H., Solomon, P. R., Doraiswamy, P. M., Smith, C. D., Jicha, G. A., Budson, A. E., Arnold, S. E., & Fadem, K. C. (2015). A clinical trial to validate event-related potential markers of Alzheimer's disease in outpatient settings. *Alzheimer's and Dementia: Diagnosis, Assessment and Disease Monitoring*, *1*(4), 387–394. https://doi.org/10.1016/j.dadm.2015.08.004

Chen, Y. T., Hou, C. J., Derek, N., Huang, S. bin, Huang, M. W., & Wang, Y. Y. (2021). Evaluation of the reaction time and accuracy rate in normal subjects, MCI, and dementia using serious games. *Applied Sciences (Switzerland)*, *11*(2), 1–14. https://doi.org/10.3390/app11020628

Christ, B. U., Combrinck, M. I., & Thomas, K. G. F. (2018). *Both Reaction Time and Accuracy Measures of Intraindividual Variability Predict Cognitive Performance in Alzheimer ' s Disease*. *12*(April). https://doi.org/10.3389/fnhum.2018.00124

Doan, D. N. T., Ku, B., Choi, J., Oh, M., Kim, K., Cha, W., & Kim, J. U. (2021). Predicting Dementia With Prefrontal Electroencephalography and Event-Related Potential. *Frontiers in Aging Neuroscience*, *13*(April), 1–19. https://doi.org/10.3389/fnagi.2021.659817

Ellen Gorus, Rudi De Raedt, Margareta Lambert, Jean-Claude Lemper, Tony Mets, M. (2008). Reaction Times and Performance Variability in Normal Aging, Mild Cognitive Impairment, and Alzheimer’s Disease. *Journal of Geriatric Psychiatry and Neurology*, *21*(3). https://doi.org/10.1177/0891988708320973

Golob, E. J., Irimajiri, R., & Starr, A. (2007). Auditory cortical activity in amnestic mild cognitive impairment: Relationship to subtype and conversion to dementia. *Brain*, *130*(3), 740–752. https://doi.org/10.1093/brain/awl375

Golob, E. J., Johnson, J. K., & Starr, A. (2002). Auditory event-related potentials during target detection are abnormal in mild cognitive impairment. *Clinical Neurophysiology*, *113*(1), 151–161. https://doi.org/10.1016/S1388-2457(01)00713-1

Luck, S. J. (2014). *An Introduction to the Event-Related Potential Technique* (2nd ed.). MIT Press.

Phillips, M., Rogers, P., Haworth, J., Bayer, A., & Tales, A. (2013). *Intra-Individual Reaction Time Variability in Mild Cognitive Impairment and Alzheimer’s Disease: Gender, Processing Load and Speed Factors*. *8*(6). https://doi.org/10.1371/journal.pone.0065712

Zurrón, M., Lindín, M., Cespón, J., Cid-Fernández, S., Galdo-álvarez, S., Ramos-Goicoa, M., & Díaz, F. (2018). Effects of mild cognitive impairment on the event-related brain potential components elicited in executive control tasks. *Frontiers in Psychology*, *9*(MAY), 1–8. https://doi.org/10.3389/fpsyg.2018.00842
